# Alexidine is a TAZ-specific small-molecule inhibitor that suppresses breast cancer invasion and metastasis

**DOI:** 10.1016/j.isci.2025.114116

**Published:** 2025-11-21

**Authors:** Anni Ge, Lishui Niu, Rachel Rubino, Kimberly Seaman, Xin Song, Yawei Hao, Kody Klupt, Natasha Iaboni, Zongchao Jia, Lidan You, Christopher J.B. Nicol, Haian Fu, Yuhong Du, Xiaolong Yang

**Affiliations:** 1 Department of Pathology and Molecular Medicine, Queen’s University, Kingston, ON K7L 3N6, Canada; 2 Department of Pharmacology and Chemical Biology, Emory University School of Medicine, Atlanta, GA 30322, USA; 3 Department of Mechanical and Industrial Engineering, University of Toronto, Toronto, ON M5S 3G9, Canada; 4 Department of Biomedical and Molecular Sciences, Queen’s University, Kingston, ON K7L 3N6, Canada; 5 Department of Mechanical and Materials Engineering, Queen’s University, Kingston, ON K7L 3N6, Canada

**Keywords:** biochemistry, biological sciences, cancer, natural sciences, pharmacology

## Abstract

Breast cancer (BC) is the most diagnosed malignancy in women and often progresses to distant metastasis. Unfortunately, current treatments inadequately address the clinical needs of metastatic BC (MBC) patients. This highlights the importance of developing effective therapies for MBC patients. One of the Hippo signaling transducers, transcriptional co-activator with PDZ-binding motif (TAZ), plays a major role in BC progression. Since TAZ mostly interacts with TEAD to facilitate its function, targeting TAZ-TEAD interaction may become a treatment approach for MBC patients. To identify inhibitors of TAZ-TEAD binding, we established a sensitive TR-FRET biosensor and performed an ultra-high throughput screen. Alexidine was identified as a TAZ-TEAD binding inhibitor capable of suppressing TAZ-induced migration and invasion in BC cells as well as metastasis in bone-on-a-chip and mouse models. In conclusion, we describe a robust method for screening inhibitors of TAZ-TEAD interaction, contributing to the development of effective cancer treatments.

## Highlights

• Developed TR-FRET and NanoLuc biosensors for TAZ inhibitor screening
• Identified alexidine as the first small molecule directly targeting TAZ
• Alexidine inhibits TAZ-driven breast cancer migration, invasion and metastasis

## Graphical abstract

**Figure:**
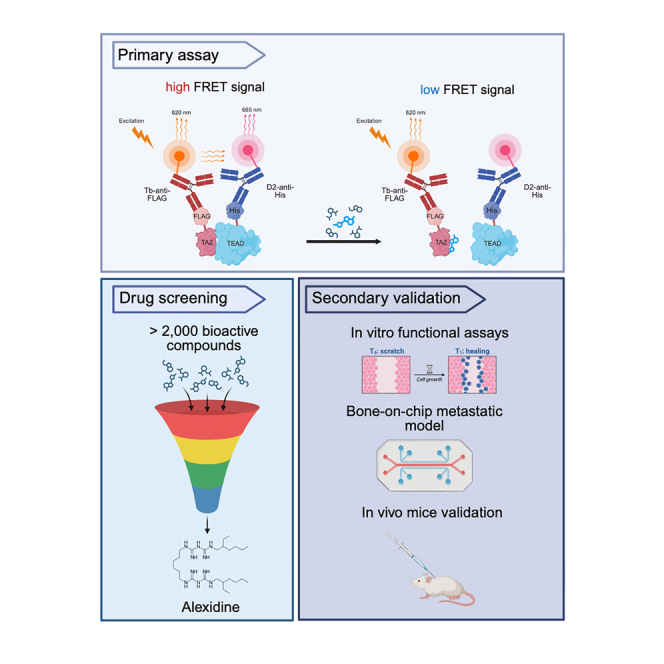

## Introduction

Breast cancer (BC) is the most common malignancy reported in women and is classified into three main subtypes based on the molecular features of the tumor, including estrogen receptor positive (ER^+^), human epidermal receptor 2 positive (HER2^+^), and triple-negative BC (TNBC; ER^−^, progesterone receptor negative [PR^−^] and HER2^−^).^1^^,^^2^ TNBC patients often exhibit basal-like tumor characteristics and suffer from poor prognosis with more aggressive phenotypes compared to non-TNBC cases.^3^^,^^4^ Both non-invasive and invasive BCs are observed among patients.^5^ Invasive BC develops when the tumor has spread beyond the ductal/glandular structures to the draining lymph nodes and other places in the breast.^5^ These invaded tumor cells can metastasize to other parts of body, including the brain, bone, lung, and liver.^6^ Metastasis accounts for over 90% of cancer-related mortalities and is the primary cause of BC fatality.^7^^,^^8^ Patients with metastatic BC (MBC) have worse clinical outcomes and a lower 10-year survival rate compared to those with localized, non-invasive tumors.^9^ The overall survival of MBC remains low due to lack of effective therapies. Moreover, the primary goal of existing treatments for MBC patients is to palliate disease symptoms and extend survival when possible.^10^ The current treatment standard for MBC is chemotherapy, which can be administered to patients differently based on the existence of hormonal receptors, metastatic state, and Oncotype DX recurrence score.^11^ Additionally, the identification of the programmed cell death protein-1 receptor (PD-1) and its ligand PD-L1 as therapeutic targets has led to the development of immune checkpoint blockade therapies.^12^ However, adverse responses and drug resistance frequently occur, impeding the establishment of effective therapies for MBC.^12^^,^^13^ Metastasis is a multistep biological process that requires tumor cells to detach from the primary site, invade and travel through circulation, and finally survive in foreign microenvironment.^7^^,^^8^ Therefore, targeting critical signaling pathways involved in metastatic events is proposed as a promising therapeutic strategy for cancer treatment development.

The Hippo pathway is a crucial signaling network for physiological development.^14^ The core components of the Hippo signaling include the mammalian sterile 20-like kinase (MST) and large tumor suppressor (LATS) kinases, which are activated in response to internal and external stimuli.^14^ The transcriptional co-activators yes-associated protein (YAP) and its paralog transcriptional co-activator with PDZ-binding motif (TAZ), are downstream targets of the Hippo kinases and phosphorylated by activated LATS, leading to their cytoplasmic sequestration or/and proteasomal degradation.^14^ Although YAP is a paralog of TAZ, previous studies provide strong evidence that TAZ, rather than YAP, plays a predominant role in breast cancer. First, TAZ is more frequently overexpressed in breast tumors than YAP.^15^^,^^16^^,^^17^ Second, TAZ is essential for maintaining stem cell-like properties and metastatic behavior in breast cancer and drug resistance, whereas YAP appears to be dispensable in this context.^15^ Third, TAZ loss, but not YAP loss, significantly reduces the expression of downstream targets such as PD-L1^18^ and CTGF,^19^ angiogenesis,^20^ and metastatic phenotypes.^21^ Altogether, these data indicate that TAZ is involved in BC metastasis and may be targeted for efficient BC treatments.

Since YAP and TAZ mainly facilitate downstream signaling through interaction with TEAD, disrupting the interaction of YAP/TAZ and TEAD complex has been recognized as an anti-cancer strategy. Notably, small-molecule inhibitors (SMIs) have been identified to target the YAP-TEAD complex, such as verteporfin (VP), the first SMI reported to suppress YAP-TEAD interaction.^22^ Since then, numerous efforts have been made to develop the protein-protein interaction (PPI) inhibitors of YAP and TEAD, including VT104,^23^ GNE-7883,^24^ and IAG933.^25^ All these inhibitors are pan-TEAD inhibitors. In contrast, research on TAZ-specific inhibitors remains limited despite its role in BC progression. Targeting TAZ may provide the following advantages: (1) Since TAZ rather than YAP is specifically associated with breast cancer metastasis and chemoresistance, targeting TAZ may provide a better strategy in treating hard-to-treat breast cancer patients; (2) selective inhibition of TAZ may reduce toxicity compared to pan-TEAD inhibition; and (3) targeting TAZ may provide a complementary strategy to existing TEAD inhibitors in the treatment of many types of cancer such as lung cancer.

High-throughput screening (HTS) coupled with bioluminescent methodologies is frequently performed in cancer studies.^26^^,^^27^^,^^28^^,^^29^ Indeed, celastrol, a YAP-TEAD inhibitor, was discovered in our previous research using a highly sensitive NanoLuc Binary Technology (NanoBiT) biosensor to monitor the interaction of YAP and TEAD in a non-invasive manner.^28^ Nevertheless, it was later realized that some drugs can randomly suppress the signal by quenching bioluminescent intensity, resulting in false positives (unpublished data). To eliminate non-specific light inhibition, we have established an orthogonal time-resolved fluorescence resonance energy transfer (TR-FRET) assay that is adapted into an ultra-high throughput screening (uHTS) format to monitor the formation of TAZ-TEAD complex. Fluorophore-conjugated antibodies are used in the TR-FRET assay detecting PPI, such as terbium (Tb) with a long emission half-life, conjugated antibody targeting one protein, which interacts with the other interacting protein recognized by another antibody conjugated with an appropriate acceptor fluorophore.^30^ The TR-FRET signal is generated and captured after donor and acceptor fluorophores are brought into close proximity by PPI.^30^ In this study, we have attempted to identify SMIs targeting TAZ and TEAD interaction through an uHTS of FDA-approved and bioactive compounds using this biosensor. Alexidine was discovered as a TAZ-specific inhibitor with anti-metastatic effect in BC. We have also reported the possible mechanism of action for alexidine in suppression of the metastatic phenotypes in TNBC.

## Results

### Generation of TAZ-TEAD biosensor for high throughput drug screen

**Figure 1:**
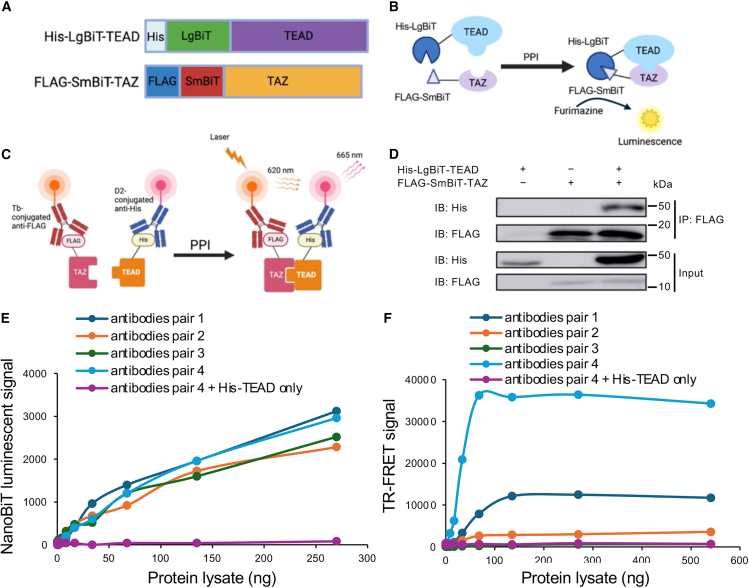
Development and validation of a TAZ-TEAD biosensor to monitor their interaction by NanoBiT and TR-FRET assays (A) Constructs of TAZ-TEAD-BS. (B) Monitoring TAZ-TEAD PPI by NanoBiT assays. (C) Monitoring TAZ-TEAD PPI by TR-FRET assays. (D) Co-IP analysis of interaction of FLAG-SmBiT-TAZ and His-LgBiT-TEAD in living cells. Plasmids expressing His-LgBiT-TEAD or FLAG-SmBiT-TAZ were transfected separately or together in HEK293T cells. Protein lysates were extracted two days after transfection with 1% NP-40 buffer. FLAG-SmBiT-TAZ was precipitated with anti-FLAG antibody, followed by western blot analysis using anti-His Antibody. The membrane is stripped and re-probed with anti-FLAG antibody. Due to increased levels of His-LgBiT-TEAD in co-transfected protein lysate (input), less amount of co-transfected lysate was used for co-IP to equalize the TEAD level. (E) Luciferase assay. (F) TR-FRET assay. Increasing protein lysates and different combined antibodies pairs (antibodies pair 1, His-Tb/Flag-D2; antibody pair 2, His-Tb-Gold/Flag-D2; antibody pair 3, His-Eu/Flag-D2; and 4, Flag-Tb/His-D2 were used in both E. and F (*n* = 1).

As previously established by Nouri et al.,^28^ a NanoLuc biosensor monitoring the binding of TAZ and TEAD1 was created with their binding domains. The TAZ-TEAD biosensor was validated both *in vitro* and *in vivo* and found to be highly sensitive and stable for HTS.^28^ To avoid non-specific bioluminescent inhibition, we further modified this biosensor by attaching His and FLAG tags into the N-terminal of LgBiT-TEAD1 and SmBiT-TAZ, respectively (Figure 1A). The modified biosensor can be used to monitor TAZ-TEAD1 protein-protein binding by both NanoLuc and TR-FRET assays (Figures 1B and 1C). To confirm that the interaction of TAZ and TEAD1 was not affected by the addition of N-terminal tags, co-IP with anti-FLAG antibody was performed using cell lysates containing His-LgBiT-TEAD1 or/and FLAG-SmBiT-TAZ. Compared to single-transfected controls, His-LgBiT-TEAD1 was only precipitated in the co-transfected sample, indicating the successful detection of TAZ-TEAD1 interaction (Figure 1D). Significantly, increases in the bioluminescent and TR-FRET signals coincided with increasing amounts of protein lysate co-expressing His-LgBiT-TEAD and FLAG-SmBiT-TAZ rather than His-LgBiT-TEAD alone (Figures 1E and 1F). These results indicate that the modified biosensor can be readily used to detect TAZ-TEAD interaction. Next, the optimal antibody pair for measuring the FRET signal was determined in the 384-well format. Four antibody pairs conjugated with different fluorophore donors or acceptors were examined. As shown in Figure 1F, high TR-FRET signal and sensitivity were achieved using the Tb acceptor-conjugated anti-FLAG antibody and D2 donor-conjugated anti-His antibody. Such antibody pair was chosen for the rest of the study. Adaptation to an ultra-high throughput format was taken place using the TAZ-TEAD biosensor to examine the reproducibility for the drug screening. The signal-to-background (S/B) ratio and *Z*-scores (*Z*′) were calculated to determine the robustness of the screening conditions. Encouragingly, substantial and comparable TR-FRET signals were observed across all replicates with high S/B ratio and *Z*′ score (Figures S1A–S1C). Overall, these results indicate the robustness of our biosensor in uHTS format monitoring TAZ-TEAD interaction.

### uHTS reveals alexidine as a small-molecule inhibitor of TAZ-TEAD interaction

**Figure 2:**
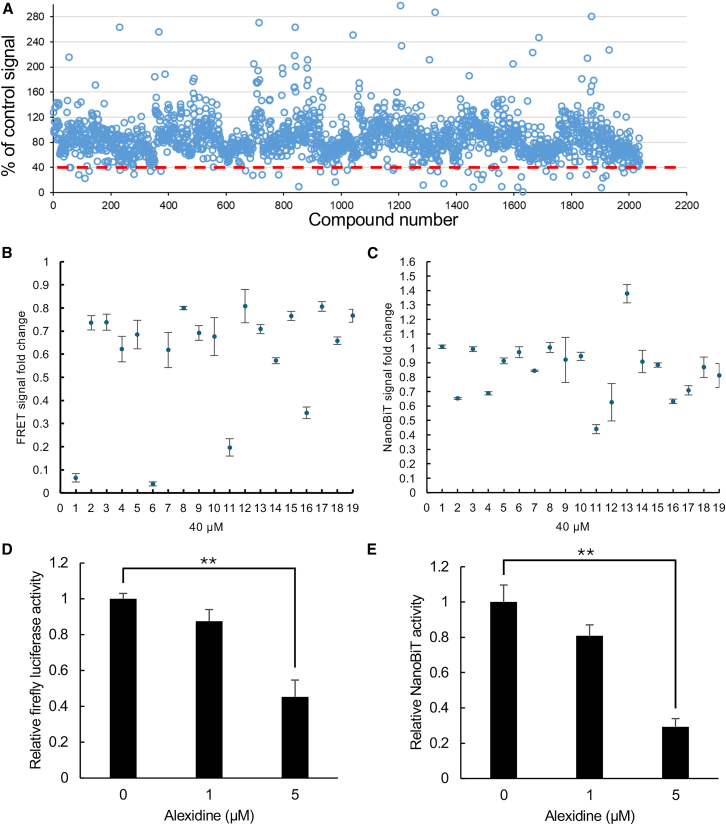
Identification of alexidine as a novel inhibitor disrupting TAZ-TEAD PPI (A) Small-scale screen of 2,036 FDA-approved and bioactive drugs by an ultra-high throughput TR-FRET assay. Compounds that showed less than 40% of the TR-FRET signal were considered the initial positive hits. The data shown are an average of 4 replicates. (B and C) Validation of positive candidates by TR-FRET (B) and NanoBiT (C) assays. Cell lysates with the TAZ and TEAD biosensor components were treated with 40 μM of each candidate for 4 h before measuring FRET or bioluminescent signals. The signal fold change was calculated based on the signal from the untreated controls. Each data point is represented as mean ± SD from biological triplicate. Functional validation of alexidine by an STBS dual luciferase reporter assay (D) Firefly luciferase assay; (E) NanoLuc luciferase assay in HEK293T cells treated with different concentrations of alexidine. The dual luciferase signals were measured using the Nano-Glo Dual Luciferase Reporter kit from Promega. The data are presented as mean ± SD from three biological replicates (*n* = 3). ∗∗*p* < 0.01 by Student’s *t* test.

With the previously validated TAZ-TEAD biosensor, we screened a library of 2,036 compounds to identify small molecules that could suppress the TR-FRET signal. The reaction buffer consisting of an equal volume of TAZ-TEAD biosensor and antibody pair was mixed with each library compound in the 1,536-well plates, which were incubated at room temperature for 2 h before measuring the TR-FRET signal. The compounds that suppressed at least 60% of the TR-FRET signal relative to no drug control were considered as initial drug candidates. Of the 2,036 drugs screened, 40 compounds displayed a substantial inhibition on TR-FRET TAZ-TEAD signal, including 22 FDA-approved molecules (Figure 2A). From here, we decided to test 19 out of the 22 FDA-approved drugs for further validating their inhibitory effects on TAZ-TEAD interaction (Table S1).

To assess the inhibitory effect of the selected candidates, each drug was subjected to additional NanoBiT and TR-FRET binding assays monitoring TAZ-TEAD interaction. Two FDA-approved drugs, alexidine and caspofungin, were subsequently identified to dramatically suppress TAZ-TEAD binding-induced signals in both assays (Figures 2B and 2C). At this stage, any drug that significantly decreased both bioluminescent and fluorescent signals was considered as a secondary positive hit. To determine whether disruption of TAZ-TEAD interaction by the secondary hits prevents TAZ-mediated downstream gene expression, these two drugs were examined by a functional reporter, called STBSs (super TEAD-binding sites) dual luciferase assay. The STBS functional reporter was created by subcloning fourteen repeats of the STBS sequence into the pNLCol2 vector expressing firefly luciferase and NanoLuc to observe TAZ and TEAD activities *in vivo*. HEK293T cells expressing such STBS dual reporter were treated with different concentrations of each secondary candidate. Alexidine, not caspofungin, was observed to significantly inhibit both firefly and NanoLuc bioluminescence at a concentration of 5 μM (Figures 2D and 2E; Figures S2A and S2B).

**Figure 3:**
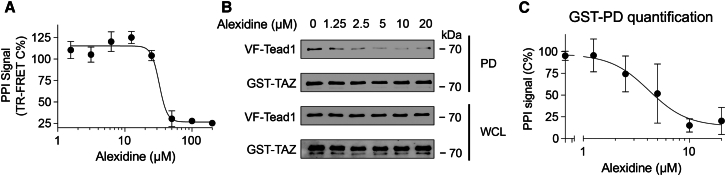
Alexidine inhibits TAZ-TEAD1 PPI in a concentration-dependent manner (A) TR-FRET dose-response curve showing alexidine dose-dependent inhibition of TAZ-TEAD1 PPI. The HEK293T cell lysates expressing His-LgBiT-TEAD1 and FLAG-SmBiT-TAZ were treated with alexidine for 18 h in a dose-response manner at the indicated concentrations. Data are presented as mean ± SD from four biological replicates. (B) Representative western blot images showing alexidine dose-dependent inhibition of TAZ-TEAD1 PPI in GST-pulldown (PD) assay. HEK293T cell lysates expressing GST-tagged TAZ and Venus-Flag (VF)-tagged TEAD1 were treated with alexidine for 18 h in a dose-response manner at the indicated concentrations and then subjected to GST pulldown assay. (C) Dose-response curve showing alexidine dose-dependent inhibition of TAZ-TEAD1 PPI. The PPI signal was quantified by gel densitometry analysis of the GST pull-down assay. Data are presented as mean ± SD from three independent experiments (*n* = 3).

To further examine if alexidine can disrupt the physical interaction of TAZ and TEAD in a direct manner, we treated HEK293T cell lysates expressing His-LgBiT-TEAD1 and FLAG-SmBiT-TAZ with increasing concentrations of alexidine for *in vitro* TR-FRET assay. Consistent with previous effect, alexidine disrupted TAZ- TEAD PPI in a dose-dependent manner (Figure 3A). In addition, a concentration-dependent inhibition of GST-TAZ and VF-TEAD1 interaction was achieved by alexidine treatment in the GST pulldown assay (Figures 3B and 3C).

**Figure 4:**
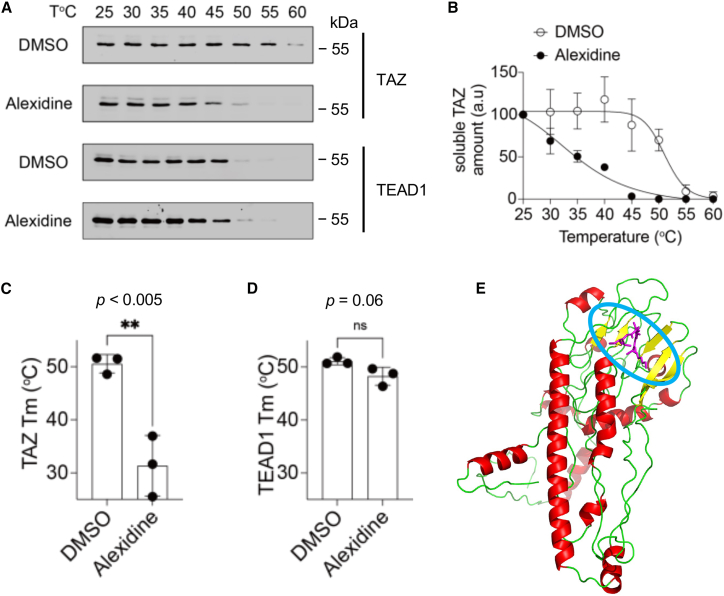
Alexidine inhibits TAZ-TEAD1 PPI by direct binding to TAZ (A) Representative western blot images showing alexidine-induced thermal stability change of TAZ and TEAD1. HEK293T cell lysates expressing TAZ and TEAD1 were treated with DMSO or alexidine (100 μM) for 4 h. After treatment, the lysates were subjected to thermal denaturation at various temperatures from 25°C to 60°C. The remaining proteins were detected by WB. (B) Thermal denaturation curves showing alexidine-induced thermal stability change of TAZ. The relative amount of soluble TAZ was quantified through gel densitometry analysis. The data are presented as mean ± SD of three independent experiments (*n* = 3). Bar graphs showing the melting temperatures of TAZ (C) or TEAD1 (D) treated with DMSO or alexidine. Tm was derived from the thermal denaturation curves. The data are presented as mean ± SD of three independent experiments (∗∗*p* < 0.005 by Student’s *t* test). (E) Structure prediction of TAZ and alexidine binding complex by Chai-1. The full-length TAZ protein sequence and SMILES code of alexidine were used to predict the complex. The blue circle highlights the alexidine binding pocket of TAZ.

Moreover, cellular thermal shift assay was conducted to demonstrate the direct interaction of alexidine with TAZ or/and TEAD *in vitro*. TAZ and TEAD thermal stability changes were measured upon alexidine binding at increasing temperature as previously described by Tang et al.^30^ A reduction in TAZ rather than TEAD stability and melting temperature (Tm) was observed in the alexidine-treated cells, compared with DMSO-treated controls (Figures 4A–4D), demonstrating that alexidine could bind to TAZ directly and reduce its stability. This observation was also supported by a molecular prediction using Chai-1, which revealed the virtual binding of alexidine to the N-terminal region (amino acids No. 33–54) of TAZ, which is located within the region (amino acids no. 27–53) essential for TAZ’s interaction with TEAD^31^ (Figure 4E).

**Figure 5:**
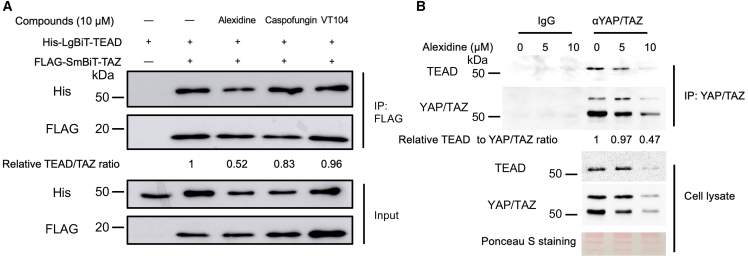
Alexidine effectively disrupts TAZ-TEAD interaction *in vivo* (A) The effect on the interaction of TAZ and TEAD biosensor in HEK293T cells treated with either alexidine, caspofungin, VT104, or DMSO control with the indicated concentration for 48 h. The TAZ-TEAD binding was detected by the presence of His-TEAD after FLAG-TAZ pull-down. The relative TEAD to TAZ ratio compared to the untreated sample was calculated through densitometry analysis in ImageJ. (B) The inhibitory effect of alexidine at different concentrations for 24 h on endogenous TAZ and TEAD binding in MDA-MB-231. The interaction of endogenous YAP/TAZ and TEAD proteins was monitored through co-immunopreciating with TEAD and YAP/TAZ using anti-YAP/TAZ antibody. Normal rabbit IgG was used as a coIP negative control. The TEAD to YAP/TAZ ratio was measured compared to the untreated control.

Co-immunoprecipitation is another common method to assess the effects of SMIs on PPI. Hence, cells co-transfected with TAZ-TEAD biosensor were treated with alexidine to test if it can inhibit TAZ-TEAD PPI *in vivo*. As shown in Figure 5A, alexidine rather than caspofungin (a negative drug control) or VT104 (a known pan-TEAD inhibitor) reduced the interaction between TAZ and TEAD in cells overexpressing the biosensor. Similar inhibition of TAZ and TEAD association was detected using MDA-MB-231 cell lysates expressing endogenous TAZ and TEAD in the presence of alexidine (Figure 5B), indicating that alexidine disrupted the interaction of TAZ and TEAD both *in vitro* and *in vivo*.

### Alexidine suppresses breast cancer cell invasion and metastasis

**Figure 6:**
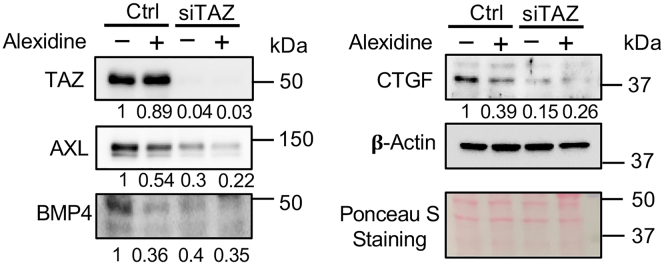
Alexidine suppresses TAZ-induced cell migration through its downstream targets in MDA-MB-231 The effect of alexidine on the expression level of TAZ-regulated targets, AXL, BMP4, and CTGF, in MDA-MB-231 with or without TAZ KD by siTAZ. The expression ratio after specific treatment is indicated below each image. The ratio is first normalized with β-actin and calculated by comparing with the untreated control. Ponceaus S staining of protein was shown to indicate equal loading of protein lysates in western blot.

Elevated TAZ activity has been observed in highly invasive BC cell lines and tissues, suggesting that it may be targeted for BC therapy.^18^^,^^21^ Importantly, TAZ in coordination with TEAD often leads to the transcription of many genes functionally important for malignant and metastatic phenotypes in BC. Thus, the functional effect of alexidine in inhibiting TAZ-TEAD interaction was characterized using high invasive breast cancer cell lines. As previously described, TAZ can facilitate its oncogenic activity by interacting with TEAD to prompt the expression of its target genes responsible for cell migration and invasion. To test whether alexidine affects TAZ signaling, we quantified protein levels of canonical TAZ-TEAD target genes AXL, BMP4, and CTGF, which promote cell migration and invasion,^32^^,^^33^^,^^34^ in MDA-MB-231 cells under control conditions (TAZ present) or after transient TAZ knockdown (KD) with small interfering RNA (siTAZ), which serves as a mechanistic benchmark. This will allow us to compare TAZ downstream target levels between wild-type and TAZ-KD cells and in cells ± alexidine to determine whether alexidine’s effects are TAZ-dependent. As expected, siTAZ reduced expression of all three targets (Figure 6). Notably, alexidine dramatically reduced the levels of AXL, BMP4, and CTGF in TAZ-wild-type cells but not in TAZ-KD cells, indicating that alexidine’s suppression of these downstream targets is indeed TAZ-dependent (Figure 6).

**Figure 7:**
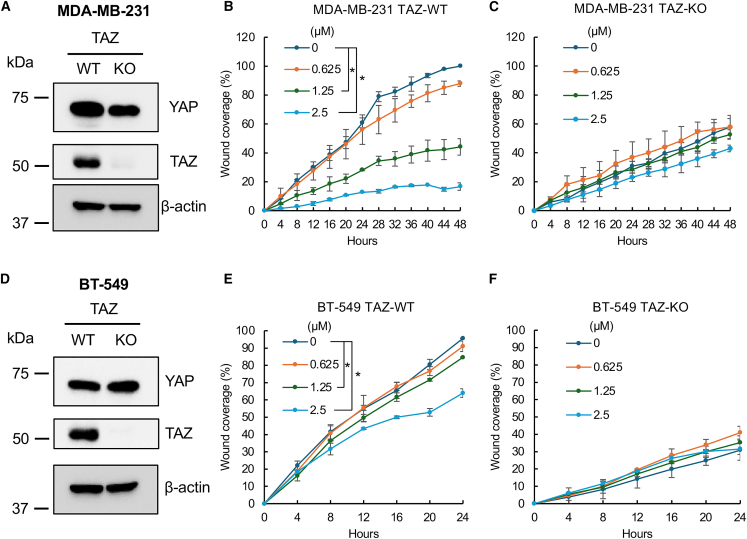
Alexidine significantly inhibited TAZ-dependent cell migration of TNBC cells Western blot analysis of TAZ and YAP expression in either WT or TAZ knockout (KO) MDA-MB-231 (A) or BT-549 (D) cells. Wound-healing cell migration analysis of WT (B and E) or TAZ KO (C and F) MDA-MB-231 (B and C) or BT-549 (E and F) cells treated with increasing concentrations of alexidine. The TNBC cells were seeded in the 96-well plates, and the wounds were made using the BioTek AutoScratch wound maker. The cells were washed with 1xPBS and supplemented with fresh media with increasing concentrations of alexidine. The wound distance was monitored every 4 h by the CELLCYTE X live cell imager. Plotted results represent the average of triplicates ±SD (*n* = 3), ∗*p* < 0.05.

Since alexidine was found to suppress TAZ-regulated genes responsible for cell migration, its effect on cell migration and invasion was determined using two TNBC cell lines, MDA-MB-231 and BT-549 with (WT) or with TAZ knockout (KO; Figures 7A and 7D). Consistent with previous data, the cell migration of MDA-MB-231 and BT-549 were suppressed significantly by alexidine in a concentration-dependent manner, whereas no major effect was observed in the parallel TAZ KO cells (Figures 7B, 7C, 7E, and 7F). Significantly, TAZ KO has no effect on YAP levels in BC cells (Figure 7A), suggesting that the loss-of-TAZ-induced decreased cell migration is independent of its paralog YAP. In addition, we also treated MCF10A non-tumorigenic mammary epithelial cells, which lack TAZ expression, with alexidine to assess its potential cytotoxicity. Treatment with alexidine concentrations up to 5 μM had minimal impact on MCF10A cell death, in contrast to the positive control, Taxol, which induced significant apoptosis at 100 nM (Figure S3). These results strongly support that the observed effects in our cell-based assays are due to specific TAZ inhibition rather than non-specific cytotoxicity. Moreover, the effect of alexidine was examined to determine whether it can affect the proliferation of breast cancer cells. Intriguingly, the cell growth of MDA-MB-231 and BT-549 was also concentration-dependently inhibited by alexidine treatment (Figures S4A and S4B), suggesting a potential of alexidine as a therapeutic agent for breast cancer.

**Figure 8:**
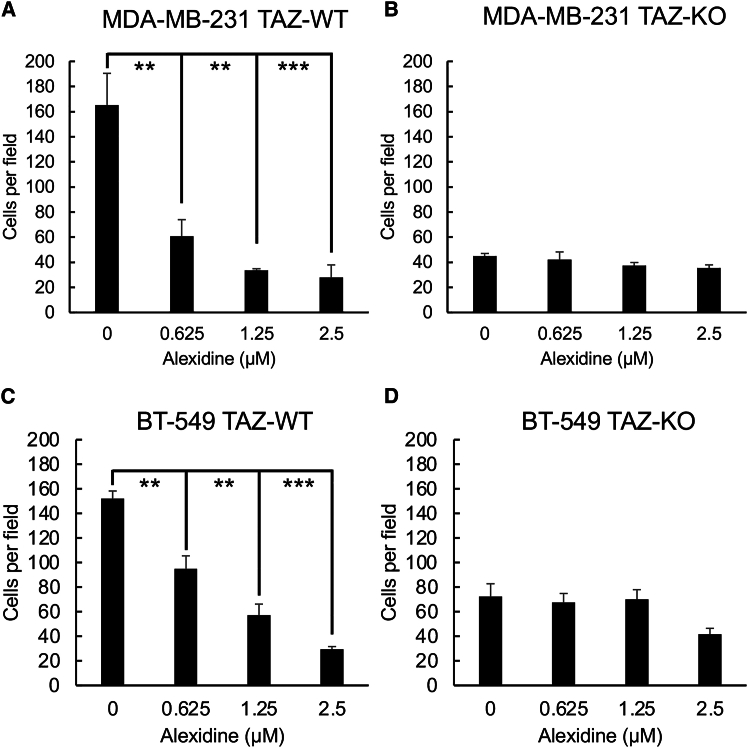
Alexidine significantly inhibited the cell invasion of TNBC cells Transwell invasion of WT or TAZ KO MDA-MB-231 (A and B) cells or BT-549 (C and D) in response to varying amounts of alexidine. TNBC cells were counted and resuspended in low serum media with varying concentrations of alexidine as indicated in the graph. Each cell suspension was dispensed into the Matrigel-coated transwell inserts within the 24-well plates, which contained the normal culture media. The number of cells invaded through the Matrigel was counted after 24 h of incubation by DAPI staining. Plotted results represent the average of biological triplicates ±SD (*n* = 3), ∗∗*p* < 0.01, ∗∗∗*p* < 0.001.

**Figure 9:**
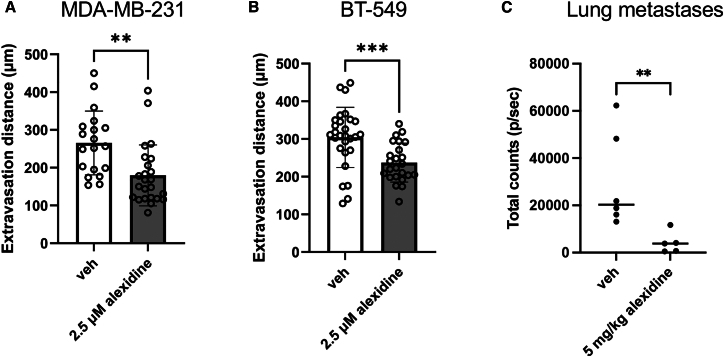
Breast cancer metastasis can be suppressed by alexidine treatment *in vitro* and *in vivo* Breast cancer cell extravasation toward bone. MDA-MB-231 (A) or BT-549 (B) cells were seeded into the lumen channel of the microfluidic device. Cancer cell extravasation distance toward the osteocyte channel was measured on day 4 after cell seeding. Specific treatment (2.5 μM alexidine or DMSO vehicle control) was introduced into the media from days 2–4 of cell seeding. The media was changed every 24 h ∗∗*p* < 0.01, ∗∗∗*p* < 0.001. Plotted data represent the results from two independent experiments (*n* = 2). (C) Breast cancer lung metastasis measured by total bioluminescence from untreated or alexidine-treated (once per day for two days at 5 mg/kg) MDA-MB-231-injected mice (*n* = 6). All data are presented as mean ± SD, ∗∗*p* < 0.01.

In addition, another critical process of cancer metastasis is the ability of tumor cells to degrade and migrate through the extracellular matrix (ECM). Thus, transwell invasion assay was performed to assess the invasive capacity of BC cells while treated with increasing concentrations of alexidine. The invasiveness of breast cancer cells through ECM was greatly impaired by alexidine in a TAZ-dependent manner (Figures 8A–8D). To further investigate the potential of alexidine in blocking the metastatic events, metastasis to bone, which is the most common metastatic site in BC, was assessed by the bone-on-a-chip model described previously by Song et al.,^35^ a physiological model mimicking *in vivo* BC metastasis, using microfluidic technologies. MDA-MB-231 and BT-549 cells were subjected to the microfluidic devices, and their extravasation distance toward osteocytes with or without alexidine was measured on day 4 after cell seeding. Consistently, reduced bone metastases were observed in both cell lines treated with 2.5 μM alexidine, compared to the vehicle control (Figures 9A and 9B). Furthermore, to investigate whether alexidine also suppresses breast cancer metastasis *in vivo*, we treated the mice with alexidine at 5 mg/kg once daily for 2 days after MDA-MB-231 cells were injected through the tail vein of each mouse. Significantly, a dramatic reduction in lung seeding was observed in alexidine-treated mice, compared to the untreated controls (Figure 9C). Overall, these data show that alexidine is a potent inhibitor of TAZ-promoted breast cancer metastases by disrupting TAZ-TEAD binding in TAZ-dependent manner. Therefore, these results show that alexidine displays promising anti-metastatic efficacy by targeting TAZ activity in BC.

## Discussion

The poor prognosis of cancer patients is often caused by the development of metastatic events, which cause most of the BC-related mortality.^7^^,^^36^^,^^37^ This risk of developing metastatic disease poses a major threat to patients’ well-being and life expectancy. Unfortunately, the high heterogeneity observed in BC patients challenges the establishment of effective treatments with low side effects. Emerging evidence reveals that the Hippo pathway plays a pivotal role in cancer progression.^14^^,^^38^ Specifically, the Hippo pathway transducers, YAP and TAZ, are transcriptional co-regulators that play a critical role in physiological functions.^14^ Dysregulation of the Hippo components can cause various diseases, and uncontrolled activity of YAP and TAZ is often required to promote different cancer hallmarks, indicating that it may be viewed as a therapeutic target.^14^^,^^15^^,^^21^ The quest to discover inhibitors of the Hippo pathway components remains a significant endeavor in cancer therapy. Many attempts have been made to directly target the formation of the YAP/TAZ-TEAD complex, revealing compounds with notable anti-tumor properties. Verteporfin is one of the well-known examples, discovered from a library of FDA-approved molecules.^22^ Its ability to inhibit YAP-TEAD interaction can suppress tumor formation in mice.^22^ However, caveats such as proteotoxic effects and poor pharmacokinetics have limited its potential as a therapeutic treatment.^39^ Similarly, other small molecules are recognized as inhibitors of TEAD palmitoylation in its central pocket that block YAP/TAZ-TEAD interaction.^23^^,^^40^^,^^41^^,^^42^ Although these TEAD inhibitors have shown strong anti-tumor potency, whether they can be successfully translated to the clinic remains to be explored.

In this study, we chose to focus on identifying SMIs of TAZ activity, given its critical and unique role in breast cancer invasion and metastasis.^15^^,^^21^^,^^43^ To achieve this, we modified a highly sensitive NanoBiT biosensor for its adaptation of the TR-FRET assay, a common method used in uHTS. The binding affinity of NanoBiT TAZ-TEAD persisted after the addition of small tags at their N-terminal domains, which allow for high throughput drug screening of PPI inhibitors. Of the 2,036 compounds screened, 19 FDA-approved drugs were considered as initial candidates, which were further validated by multiple biochemical assays to eliminate false positives caused by quenching effect in fluorescent signals. Among the positive hits, alexidine was finally verified to inhibit TAZ-TEAD interaction through *in vitro* cell-free and *in vivo* cell-based assays. Although the effective concentration of alexidine differed between *in vitro* binding assays and *in vivo* functional studies, this discrepancy may be attributed in part to the higher protein content present in the purified fusion proteins used for the cell-free assays compared to the cell-based experiments, necessitating a higher concentration of alexidine to effectively target TAZ activity in the *in vitro* setting. Additional factors likely contribute to this shift, including differences in assay conditions (e.g., prolonged incubation times and buffer compositions in cell-free formats that may affect compound solubility or stability), intracellular accumulation of the drug, enhanced cell permeability, and local concentration effects in cellular compartments, or physiological conditions that amplify effective exposure at the target site in cells.^44^^,^^45^^,^^46^^,^^47^ Such variations in potency between biochemical and cellular assays are commonly reported for PPI inhibitors, where cell-free systems often underestimate cellular efficacy due to the absence of physiological context (e.g., as observed with some YAP-TEAD disruptors like verteporfin and celestrol).^22^^,^^28^ Future studies employing TR-FRET with fully purified TAZ and TEAD proteins would provide a more precise determination of the intrinsic binding affinity of alexidine and help dissect these contributions. Hence, a higher alexidine concentration might be needed to efficiently target TAZ activity in the *in vitro* binding assays. Even though the antimicrobial ability of alexidine and other analogs has been reported,^48^ very little is known about its anti-tumor effect in BC. Herein, we describe that alexidine functions as a TAZ-TEAD binding inhibitor that suppresses TAZ-dependent metastatic phenotypes, including cell migration/invasion, bone metastasis in bone-on-a-chip system, and lung metastasis in mouse model. Furthermore, even though the exact molecular mechanism needs further exploration, our findings suggest that alexidine may directly bind to the N-terminal region of TAZ and reduces its stability (Figure 4). We speculate that alexidine binding to TAZ may change its 3D conformation, which increases the susceptibility to proteosome-mediated protein degradation as described previously.^49^^,^^50^^,^^51^^,^^52^

In summary, we have successfully established a TR-FRET assay coupled with NanoBiT biosensor for screening SMIs of TAZ-TEAD interaction. Alexidine is discovered from the screening and may be applied as a potential anti-metastatic agent in BC. This study hence provides preliminary data and serves as a proof-of-concept research in repurposing FDA-approved compounds for anti-cancer treatments.

### Limitations of the study

We recognize that there are potential limitations to this study. While we have screened over 2000 compounds using a custom library, a larger set of drugs may be included in the future to discover more TAZ-specific inhibitors. Moreover, given the difficulties in predicting the binding pocket of TAZ, we have employed an alternative tool to explore the binding between alexidine and TAZ. Further exploration and validation are thus needed to observe the exact interaction complex through other biochemical assays, such as surface plasmon resonance or TR-FRET with fully purified proteins, to precisely define the binding affinity of alexidine to TAZ. In addition, the efficacy of alexidine in reducing TAZ-regulated metastatic events requires careful evaluation in appropriate *in vivo* animal models. Finally, chemical modification of alexidine may be needed to eliminate off-target effects and increase specificity toward TAZ.

## Resource availability

### Lead contact

Further information and requests for resources and reagents should be directed to and will be fulfilled by the lead contact, Xiaolong Yang (xiaolong.yang@queensu.ca).

### Materials availability

Plasmids generated in this study are available upon request to the lead contact. Other materials are commercially available.

### Data and code availability

• All data reported in this study is available from the lead contact upon request.
• This paper does not report original code.
• All additional information required to reanalyze the data in this study will be made available from the lead contact upon request.

## Acknowledgments

The authors thank Dr. Fu’s team for performing uHTS at Emory University, Dr. You’s team at University of Toronto for the bone metastatic study as well as Dr. Nicol’s team for *in vivo* mice work. This study was funded by the 10.13039/501100000024Canadian Institutes of Health Research, grant numbers 186142 and 148629 to X.Y. This work is also in part supported by the National Cancer Institute’s Emory Lung Cancer SPORE (P50CA217691 to H.F.) and the NCI Emory Lung Cancer Program Project (P01CA257906 to H.F.).

## Author contributions

Conceptualization, H.F., Y.D., and X.Y.; methodology, A.G., L.N., Y.H., K.K., Z.J., and Y.D.; validation, A.G., L.N., Y.D., K.S., N.I., X.S., and R.R.; writing – original draft preparation, A.G., L.N., K.K., Y.D., K.S., X.S., and X.Y.; writing – review and editing, A.G., Z.J., H.F., Y.D., and X.Y.; visualization, A.G., L.N., Y.D., K.S., and X.S.; supervision, H.F., Y.D., Z.J., C.J.B.N., L.Y., and X.Y.; project administration, X.Y.; funding acquisition, H.F., Y.D., and X.Y. All authors have read and agreed to the published version of the manuscript.

## Declaration of interests

The authors declare no competing interests.

## STAR★Methods

### Key resources table

**Table:** 

REAGENT or RESOURCE	SOURCE	IDENTIFIER
**Antibodies**		

Tb cryptate-conjugated FLAG (M2) mouse mAb antibody	Cisbio Bioassays	Cat# 61FG2TLB; RRID: AB_2884026
Tb cryptate-conjugated 6His mouse mAb antibody	Cisbio Bioassays	Cat# 61HISTLF; N/A
Tb cryptate Gold-conjugated 6His mAb antibody	Cisbio Bioassays	Cat# 61HI2TLF; RRID: AB_3676734
Eu cryptate-conjugated 6His mAb antibody	Cisbio Bioassays	Cat# 61HISKLA; N/A
D2-conjugated 6His mouse mAb antibody	Cisbio Bioassays	Cat# 61HISDLF; RRID: AB_2884027
D2-conjugated FLAG (M2) mouse mAb antibody	Cisbio Bioassays	Cat# 61FG2DLF; N/A
FLAG (M2) mouse mAb antibody	Sigma-Aldrich	Cat# F1804; RRID: AB_262044
His (HIS.H8) mouse mAb antibody	Abcam	Cat# ab18184; RRID: AB_444306
YAP/TAZ (H-125) rabbit pAb antibody	Santa Cruz	Cat# sc-15407; RRID: AB_2273277
TAZ mouse mAb antibody	BD Biosciences	Cat# 560235; RRID: AB_1645338
TAZ (E8E9G) rabbit mAb antibody	Cell signaling	Cat# 83669; RRID: AB_2800026
TEAD1-4 (EPR15629) rabbit mAb antibody	Abcam	Cat# ab197589; N/A
TEAD1 (D9X2L) rabbit mAb antibody	Cell signaling	Cat# 12292; RRID: AB_2797873
AXL (C89E7) rabbit mAb antibody	Cell Signaling	Cat# 8661; RRID: AB_11217435
BMP4 mouse mAb antibody	R&D systems	Cat# MAB757; RRID: AB_2063679
CTGF rabbit pAb antibody	Abcam	Cat# ab6992; RRID: AB_305688
β-actin mouse mAb antibody	Sigma-Aldrich	Cat#A5441; N/A

**Chemicals, peptides, and recombinant proteins**		

Acetic acid	Sigma-Aldrich	Cat# A6283
NaOH	Sigma-Aldrich	Cat# BDH7247-1
1× DPBS	Sigma-Aldrich	Cat# D8537-500 ML
5× DPBS	Sigma-Aldrich	N/A
Fibronectin	Sigma-Aldrich	Cat# F1141-2 MG
Type-1 rat-tail collagen	Corning	Cat# 354236
Alexidine dihydrochloride	TargetMol	Cat# T14177; PubChem CID: 102678
Caspofungin Acetate	TargetMol	Cat# T1799; PubChem CID: 16119813
VT104	Aobious INC	Cat# AOB12135; PubChem CID: 146909371
Taxol (Paclitaxel)	Sigma	Cat# T7402; PubChemCID: 36314
Clarity^TM^ Western ECL Substrate	BIO-RAD	Cat# 1705060
Dynabeads^TM^ Protein G for co-IP	Thermo Fisher Scientific	Cat# 10003D
Glutathione Sepharose beads	GE	Cat# 17075605
Nano-Glo Live substrate	Promega	Cat# N113A
Mounting Medium with DAPI − Aqueous, Fluoroshield	Abcam	Cat# ab104139
Matrigel	Corning	Cat# 354230
Trypan blue solution, 0.4%	Sigma-Aldrich	Cat# T8154-100 ML

**Critical commercial assays**		

Nano-Glo Dual Luciferase reporter assay kit	Promega	Cat# N1640

**Experimental models: Cell lines**		

Human: HEK293T	Laboratory of Dr David Lillicrap	N/A
Human: MDA-MB-231	ATCC	HTB-26
Human: MDA-MB-231-TAZ KO	Laboratory of Dr Xiaolong Yang	N/A
Human: BT-549	ATCC	HTB-122
Human: BT-549-TAZ KO	Laboratory of Dr Xiaolong Yang (mycoplasma-free)	N/A
Human: MCF10A	ATCC (mycoplasma-free)	CRL-10317
Murine: MLO-Y4	Laboratory of Dr Lynda Bonewald (mycoplasma-free)	N/A
Human: HUVEC	Laboratory of Dr Craig Simmons (mycoplasma-free)	N/A

**Experimental models: Organisms/strains**		

Mouse: Balb/c−*Rag2*^−/−^*IL2rg*^−/−^	Laboratory of M. Ito	N/A

**Recombinant DNA**		

His-LgBiT-TEAD1/pcDNA3.1	Nouri et al.^28^	N/A
FLAG-SmBiT-TAZ/pcDNA3.1	This paper	N/A
STBS/pNLCol2	This paper	N/A
GST-TAZ	This paper	N/A
Venus-FLAG-TEAD1	This paper	N/A

**Software and algorithms**		

ImageJ 1.53	Schneider et al.^7^	https://imagej.nih.gov/ij/
Prism 10	GraphPad	https://www.graphpad.com/scientific-software/prism/
CELLCYTE STUDIO	CYTENA	N/A
Chai-1		https://lab.chaidiscovery.com
Living Image 4.8.2	Revvity	https://www.revvity.com/ca-en/software-downloads/in-vivo-imaging

### Experimental model and study participant details

#### Cell lines

The human MCF10A, MDA-MB-231 and BT-549 cell lines were purchased from ATCC. The human embryonic kidney HEK293T, umbilical vein endothelial cells (HUVECs) and murine osteocyte-like MLO-Y4 cell line were gifted from Dr. David Lillicrap (Queen’s University, Canada), Dr. Craig Simmons (University of Toronto, Canada) and Dr. Lynda Bonewald (Indiana University, USA), respectively. The stable TAZ knockout (KO) cell lines were created using similar methods as previously described by Rensburg et al.^18^ Briefly, stable TAZ knockout was achieved in MDA-MB-231 and BT-549 by transducing lentiCRISPRv1 plasmids containing CRISPR-Cas9 TAZ KO constructs. sgRNA sequences are listed in the Supplementary data. MDA-MB-231 and HEK293T cells were cultured in Dulbecco’s Modified Eagle’s Medium (DMEM, Sigma-Aldrich) containing 10% fetal bovine serum (FBS, Sigma-Aldrich), and 1% penicillin/streptomycin (P/S, Invitrogen). BT-549 cells were maintained in RPMI-1640 Medium (Sigma-Aldrich) containing 10% FBS, 1% P/S, and 0.023 U/ml insulin. MLO-Y4 cells were grown in 94% v/v α-MEM basal media (Gibco), supplemented with 2.5% FBS (Gibco), 2.5% calf serum (Gibco), and 1% P/S (Gibco) on culture dishes pre-coated with 0.15 mg/mL type-1 rat-tail collagen (Corning) in 0.02N acetic acid (Sigma-Aldrich). HUVECs were cultured in EndoMax basal medium (Wisent) supplemented with 2% EndoMax growth supplement (Wisent), 10% FBS, and 1% P/S. All cell lines were maintained at 37 °C with 5% CO_2_ and tested negative for mycoplasma contamination by PCR.

#### Mice

Four to six weeks old of immunodeficient Balb/c female mice were used to study breast cancer metastatic events *in vivo*. Breeding pairs of *Rag2*^−/−^*IL2rg*^−/−^ double knockout mice (alymphoid) Balb/c mice were kindly provided by Dr. M. Ito (Central Institute for Experimental Animals, Kawasaki, Japan), and animal were maintained in-house at the animal facility at Queen’s University. Each drug dosage used in the study with was normalized based on the weight of individual mouse. All animal procedures were followed in accordance with Canadian Council on Animal Care guidelines and were approved by the Queen’s University Animal Care Committee (Protocol#2025–2599).

### Method details

#### TAZ-TEAD biosensor plasmid generation

The FLAG-tagged interacting domains of TAZ (aa 13–119) and His-tagged TEAD1 (aa 194–411) were fused with SmBiT and LgBiT, respectively, by overlapping PCR using full-length TAZ (accession number NM_001168278.2), TEAD1 (accession number NM_021961.6), and NanoLuc as templates. The PCR products were digested by BamHI/NotI and subsequently subcloned into pcDNA3.1/hygro vector.

#### uHTS TR-FRET screening

uHTS TR-FRET was performed at the Emory University School of Medicine. The screening conditions were modified based on a previous publication.^30^ Briefly, HEK293T cells were transfected with biosensor plasmids mentioned in the previous section. Cell lysates extracted from HEK293T cells expressing FLAG-LgBiT-TEAD1 and His-SmBiT-TAZ were combined with an equal volume of optimal antibody pair in the black 1536-well plate (Corning) with a final volume of 5 μL per well. The optimal amount of cell lysates and antibodies utilized were adjusted proportionally based on the conditions from the 384-well plate. The last column was used as empty background control. Next, the 2036 Emory Enriched Bioactive Library (EEBL) compounds were added into each well for each plate using Biomek NXP Automated Workstation (Beckman) from a compound stock plate to achieve a final concentration of 40 μM. After 2 h of incubation at room temperature, the FRET signal was captured using the BMG Labtech PHERAstar FSX reader with the HTRF optic module. The assay performance for uHTS was evaluated by Signal-to-background (***S***/*B*) ratio and ***Z***′ score according to the following equations:

S/B=FRETppi/FRETvector

$$Z^{\prime }=1-\left(3\times SDppi+3\times SDvector\right)/\left(FRETppi-FRETvector\right)$$

where ***FRETppi*** and ***FRETvector*** are the FRET signals from lysates with either biosensor components or blank controls. ***SDppi*** and ***SDvector*** are the standard deviations of replicates. The signal window and robustness of the TR-FRET assay were revealed by ***S***/*B* ratio and ***Z***′, respectively. A ***Z***′ score of 0.5–1 indicated the robustness of the assay for HTS.

In the case of the TR-FRET assay performed for primary validation, cell lysates with the same biosensor were incubated with 40 μM of each initial drug candidate in the 96-well plates for 2 h at 4°C. Next, a mixture of previously optimized antibody pair was added to each well for an additional 2-h incubation before the FRET signal was measured with the FLUOstar Omega plate reader.

#### NanoBiT assay

NanoBiT assay was performed as described previously.^27^^,^^28^^,^^29^ In brief, HEK293T cells were transfected with FLAG-SmBiT-TAZ and His-LgBiT-TEAD1 plasmids using PolyJet transfection reagent (SignaGen, Rockville, MD, USA). The cell lysates were extracted using 1× passive lysis buffer (PLB; Promega, WI, USA) after 24 h post-transfection. For initial validation, the cell lysates were added in 96-well plates and incubated with 40 μM of each positive hit on a shaker for 4 h at 4°C. The bioluminescent signals were measured with Nano-Glo Live substrate (1:500 dilution from stock, Promega) using the GloMax Navigator Microplate Luminometer (Promega).

#### STBS functional reporter assay

The sequences of Super TEAD Binding Sites (STBS) were subcloned into the pNLCol2 vector (Promega), which could express firefly luciferase (FLuc) and NanoLuc at the same level mediated by the P2A sequence. The addition of the STBS within this vector allowed the expression of FLuc and NanoLuc enzymes to be induced by TAZ and TEAD activities. HEK293T cells expressing the STBS dual luciferase reporter were seeded to the 96-well plates and treated with varying concentrations of alexidine or caspofungin for 24 h in the incubator. The next day, the cells were lysed with 10 μL of 1× PLB per well and incubated on a shaker at 200 rpm for 15 min at room temperature. The firefly and NanoLuc bioluminescent signals were measured sequentially using the Nano-Glo Dual Luciferase Reporter assay kit (Promega) following the manufacturer’s instruction.

#### GST pull-down (PD) assay

HEK293T cells overexpressing GST-TAZ or Venus-Flag-TEAD1 (VF-TEAD1) were lysed using NP-40 lysis buffer containing 1% NP-40, 20 mM Tris (pH 8.0), 137 mM NaCl, 5% glycerol, along with protease inhibitors (Sigma-Aldrich) and phosphatase inhibitors (Sigma-Aldrich). The resulting lysates were incubated with various concentrations of alexidine for 18 h at 4 °C before they are incubated with glutathione-conjugated beads (GE) for 2 h at 4 °C. After incubation, the beads were washed three times with 1% NP-40 buffer, and the bound proteins were eluted using 2× Laemmli sample buffer (BIO-RAD). Expression levels of VF-TEAD1 and GST-TAZ were detected by western blot analysis using rabbit monoclonal anti-TAZ (E8E9G) antibody and rabbit monoclonal anti-TEAD1 (D9X2L) antibody, respectively. GraphPad Prism was used to fit the data and calculate the percentage (%) of PPI signal.

#### Cellular thermal shift assay (CETSA)

The endogenous TAZ and TEAD1 protein stability was assessed using the CETSA method by treating HEK293T cell lysates with alexidine at the desired concentrations. Briefly, HEK293T cells were harvested and resuspended in 1 mL 1× PBS containing protease inhibitors and phosphatase inhibitors. Cell lysis was performed by subjecting the cell suspension to three freeze-thaw cycles, alternating between liquid nitrogen and 25 °C. The lysates were then treated with DMSO or alexidine for 4 h at 4 °C. Following treatment, aliquots of the lysate (95 μL/tube) were transferred to PCR tubes for thermal denaturation, carried out at temperatures ranging from 25°C to 60 °C in a thermal cycler for 5 min. After thermal treatment, samples were centrifuged at 20,000 g for 20 min at 4 °C to pellet cell debris and precipitated or aggregated proteins. The soluble proteins were subsequently analyzed by SDS-PAGE and Western blotting analysis of TAZ and TEAD1 using rabbit monoclonal anti-TAZ (E8E9G) antibody and rabbit monoclonal anti-TEAD1 (D9X2L) antibody, respectively. The intensity of western blot bands was quantified as described in *2.11*. GraphPad Prism was used to fit the data and identify the temperature corresponding to 50% soluble protein as the melting temperature (Tm).

#### Structure prediction of TAZ-alexidine binding

The binding complex of TAZ and alexidine was predicted using Chai-1 by providing the full-length protein sequence of TAZ and SMILES code of alexidine. Multiple sequence alignment was included, and no restraints were introduced.

#### Protein extraction, western blot analysis and co-IP

Except mentioned otherwise, the protein lysates were extracted after the cells were grown to at least over 80% confluent using RIPA (50 mM Tris HCl, 150 mM NaCl, 1.0% (v/v) NP-40, 0.5% (w/v) Sodium Deoxycholate, 1.0 mM EDTA, 0.1% (w/v) SDS and 0.01% (w/v) sodium azide, pH = 7.4) with the Complete EDTA-free protease inhibitor cocktail tablet (Roche). If the lysates were subjected to co-IP, 1% NP-40 buffer was used instead. The supernatant was obtained by centrifuging the cell lysates at 12,000g at 4°C for 10 min, and the protein concentration was determined using the DC protein assay kit (BIO-RAD). Western blot (WB) analysis was performed with 10 μg of protein lysates for each sample after it was boiled with 1× loading buffer at 95°C for 5 min. The proteins were then resolved by SDS-PAGE (10–12%) and transferred to Hybond-ECL nitrocellular membrane (Amersham), which was blocked by Everyblot blocking solution (BIO-RAD) for 5 min at room temperature. The membrane was washed with 1× TBST buffer twice before it was probed with primary antibodies at room temperature for 1 h or at 4°C overnight. Secondary antibodies (1:2500 dilution) were added and incubated for 15 min at room temperature after the membranes were rinsed and washed with 1× TBST buffer twice. The proteins were detected by chemiluminescence reagent (BIO-RAD). Antibodies and their corresponding dilutions are as following: mouse monoclonal anti-FLAG (F1804, Sigma, 1:1,000) antibody; anti-His (1:1,000); rabbit polyclonal anti-YAP (sc-15407, Santa Cruz H125, 1:1,000); mouse monoclonal anti-TAZ (560235, BD Biosciences, 1:1,000); anti-TEAD (1:1,000); mouse monoclonal anti-β-actin (A5441, Sigma, 1:1,000).

For co-immunoprecipitation, HEK293T cells were transfected with plasmids expressing either His-LgBiT-TEAD1 or FLAG-SmBiT-TAZ. After 5 h post-transfection, cells were counted and seeded into the 6-well plate. The next day, the seeded cells were treated with different compounds (DMSO, alexidine, caspofungin, or VT104) at 10 μM for 48 h. Cell lysates were then extracted using 1% NP-40 lysis buffer with protease inhibitor. Two μg of anti-FLAG antibody was added to 500 μg of cell lysates, which were incubated overnight at 4°C. The next day, 20 μL of protein A magnetic beads was added to the tube, which was incubated at 4°C for 2 h. The samples were then washed three times with 1% NP-40 buffer before the immunoprecipitated proteins were eluted by boiling with 2× SDS buffer and subjected to WB.

For co-IP with endogenous TAZ and TEAD, MDA-MB-231 cells were treated with increasing concentrations of alexidine for 24 h before the cell lysates were obtained with 1% NP-40 lysis buffer. Two μg of anti-YAP/TAZ antibody or normal IgG as the negative control was added to each tube with 1 mg of cell lysates for each sample. The tubes were incubated overnight at 4°C, followed by the addition of protein A magnetic beads for an additional 2-h incubation. The samples were washed three times with 1% NP-40 buffer and boiled with 2× SDS buffer for subsequent WB analysis.

#### Wound healing assay

Approximately 5x10^4^ BT549 and MDA-MB-231 cells were seeded in 96-well plates. The next day, the wounds were introduced using BioTek AutoScratch wound maker (Agilent, CA, USA). Each well was washed with 1× PBS, and fresh media with increasing concentrations of alexidine was added into each well. The wound distance was recorded every 4 h using CELLCYTE X live cell imager (CYTENA, USA) until the scratch closed completely.

#### Transwell migration assay

Approximately 1x10^5^ BT549 and MDA-MB-231 cells suspended in low-serum media (1% FBS) were seeded into the upper chamber of the Matrigel-coated transwell inserts (8 μM; BD bioscience) from the 24-well plates, which contained normal culture media in the lower compartment. Approximately 24 h later, the low-serum media and Matrigel were removed, and the invaded cells were fixed with 70% ethanol for 10 min at room temperature. The cells were stained using DAPI containing mounting solution (Abcam) after they were air dried for about 10–15 min. Images were captured using a Nikon TE-2000U (Melville, NY, USA) inverted microscope with 10× magnification.

#### Trypan blue exclusion analysis of cell viability

The non-tumorigenic breast cancer cell line, MCF10A, was seeded in triplicates in 24-well plates at 3.5 ×10^4^ cells per well. The next day, alexidine or paclitaxel was added at increasing concentrations to each well. After 48 h of drug treatment, cell viability was determined by trypan blue, which stains dead cells. The mean and standard deviation of the percentage of cell death for each well was calculated based on the number of live and dead cells.

#### Cell proliferation assay

Breast cancer cells, including BT549 and MDA-MB-231, were seeded in the 24-well plates with a concentration of 2×10^4^ cells per well. The next day, fresh culture media supplemented with varying concentrations of alexidine was introduced to each well, and the cell confluency for eahc well was monitored by CELLCYTE X live cell imager every 1 h for 3 days.

#### Microfluidic analysis of breasts cancer cell extravasation

The microfluidic analysis of breast cancer cell extravasation was performed as described previously.^35^ The microfluidic devices were disinfected with 70% ethanol and rinsed with 1× DPBS (Sigma-Aldrich). The osteocyte channels were coated with 0.15 mg/mL type-1 rat-tail collagen for 1 h at room temperature. All osteocyte, side, and lumen channels were coated with 100 μg/mL fibronectin (Sigma-Aldrich) in 1× DPBS for 40 min at 4 °C. The hydrogel solution with 5.5 mg/mL type-1 rat-tail collagen and 2.5 mg/mL Matrigel was prepared with 5× DPBS (Sigma-Aldrich), 5N NaOH (Sigma-Aldrich), 11.07 mg/mL of type-1 rat-tail collagen and 8.5 mg/mL Matrigel on ice. The prepared hydrogel was loaded slowly to the lumen channel and removed within 30 s. The coated device was incubated in the 37 °C incubator to allow hydrogel solidification. All channels were filled with pre-warmed media after gelation. Each side of lumen channel was seeded with 2000K HUVECs/mL at different orientations to coat the bottom layer and left-tilting layer of the lumen channel. The osteocyte channel was seeded with 1500K MLO-Y4 cells/mL. After the seeded HUVECs and MLO-Y4 cells had attached, fluorescent-labeled MDA-MB-231 or BT549 cells (CellTracker Green) were seeded at 4000K/mL into the bottom layer and left-tilting layers of the lumen channel. Media in the lumen channels were prepared with 1:1 mixture of HUVEC and MDA-MB-231 growth media. The osteocyte channel was filled with MLO-Y4 growth media. Treatment (2.5 μM alexidine or DMSO vehicle control) was included in the microfluidic media into the lumen channels starting from days 2–4 of the experiment. Media from both channels were replenished every 24 h. Cancer cell extravasation was imaged with a fluorescent microscope on days 1 and 4 after seeding. Extravasation distance was quantified by measuring the location changes of cancer cells on day 4 using ImageJ. Five side channels were in one microfluidic device, and six devices were on the microfluidic platform. The extravasation distance in each side channel was counted as one data point on the graph.

#### *In vivo* mouse model of lung metastases

A million of MDA-MB-231 cells expressing firefly luciferase pre-treated without or with 5 μM alexidine for 48 h were suspended in ice-cold PBS and injected into the tail vein of 4–6 weeks old female Balbc−*Rag2*^−/−^*IL2rg*^−/−^ mice (*n* = 3/group). Subsequently, each mouse was injected i.p. without or with 5 mg/kg of alexidine daily for two days. After 48 h of initial injection, bioluminescence imaging was performed using the IVIS Lumina Series III *in vivo* imaging system (PerkinElmer) with 150 mg/kg D-luciferin in PBS to measure lung metastases. The lung seeding was quantified as total counts (photon/sec) in Living Image software.

#### Densitometric analysis by ImageJ

To quantify the protein bands, WB images were imported and analyzed using the Gel Analyzer tool of ImageJ (NIH, Version 1.53t). The relative ratio was calculated after the band intensity was subtracted from the background signal.

### Quantification and statistical analysis

Statistical analysis was conducted using the two-tailed Student’s *t* test in GraphPad Prism software version 10. A *p*-value of less than 0.05 was considered statistically significant.

## Supplemental information

Document S1. Figures S1−S5
