# Supplementary material for: Alexidine is a TAZ-specific small-molecule inhibitor that suppresses breast cancer invasion and metastasis
Source: iScience. 2025 Nov 21;28(12):114116. doi: 10.1016/j.isci.2025.114116 (PMC12719775; doi:10.1016/j.isci.2025.114116)
Supplement: Document S1. Figures S1−S5 [file mmc1.pdf]

iScience, Volume *28*

Supplemental information

Alexidine is a TAZ-specific small-molecule inhibitor that suppresses breast cancer invasion and metastasis

Anni Ge, Lishui Niu, Rachel Rubino, Kimberly Seaman, Xin Song, Yawei Hao, Kody Klupt, Natasha Iaboni, Zongchao Jia, Lidan You, Christopher J.B. Nicol, Haian Fu, Yuhong Du, and Xiaolong Yang

Supplementary figures (alexidine)

Ge et al.

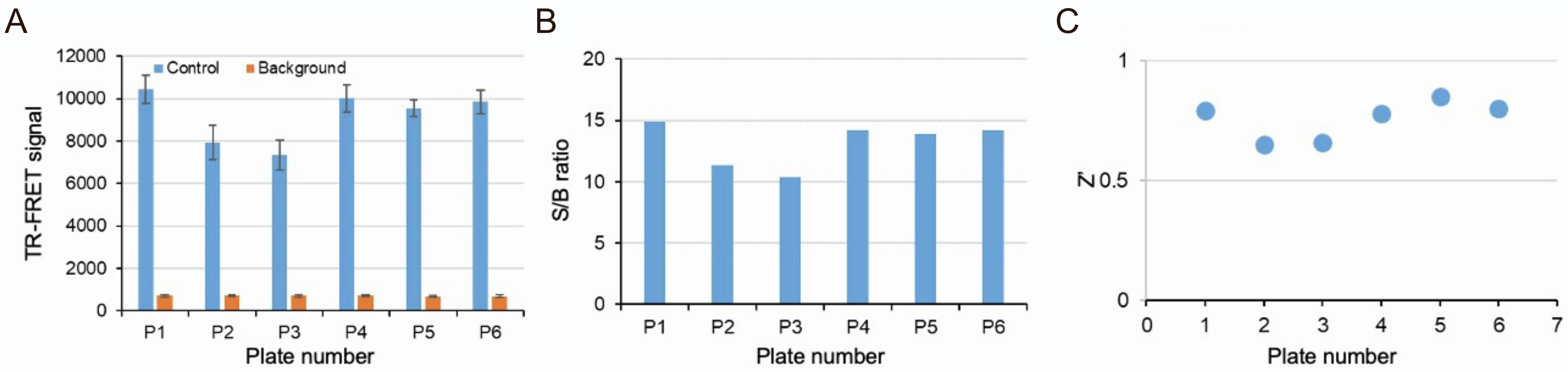

Figure S1. Optimization of TR-FRET assay in high-throughput format. A-C. Assay miniaturization into 1536-well format. The TR-FRET signal was measured with a Multilabel plate reader. Six parallel screening plates were performed to ensure the reproducibility of the screening. The results shown are an average of 32 replicates, and the background indicates the signal from TR-FRET antibodies in the absence of cell lysate (n = 32).

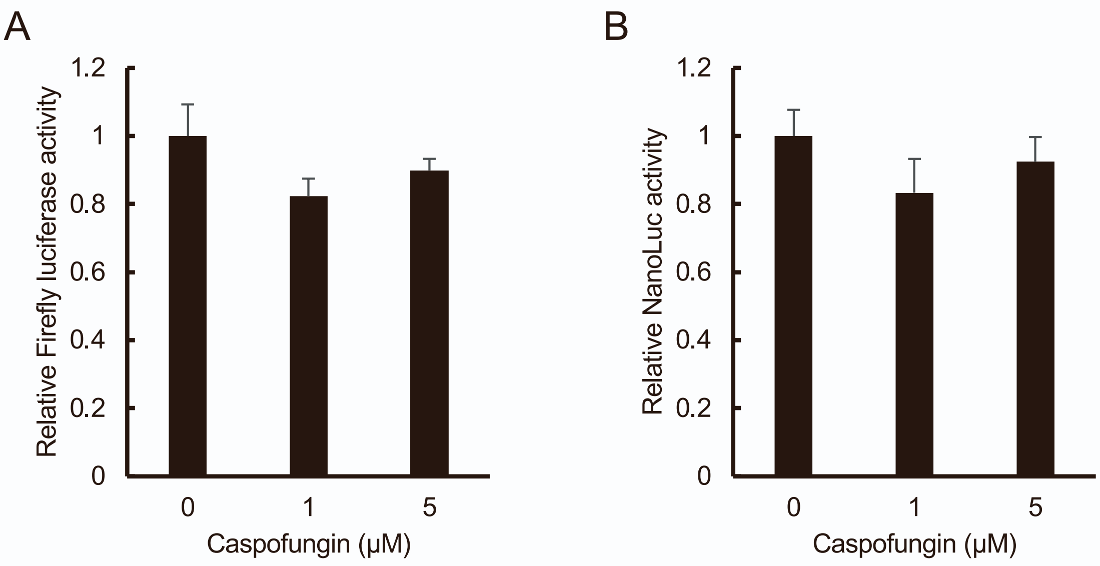

Figure S2. Functional validation of caspofungin by the STBS functional assay. Caspofungin was examined by a functional reporter assay through TAZ-dependent firefly luciferase (A) and NanoLuc (B) activities. All data are shown as the average of triplicate + SD (n = 3).

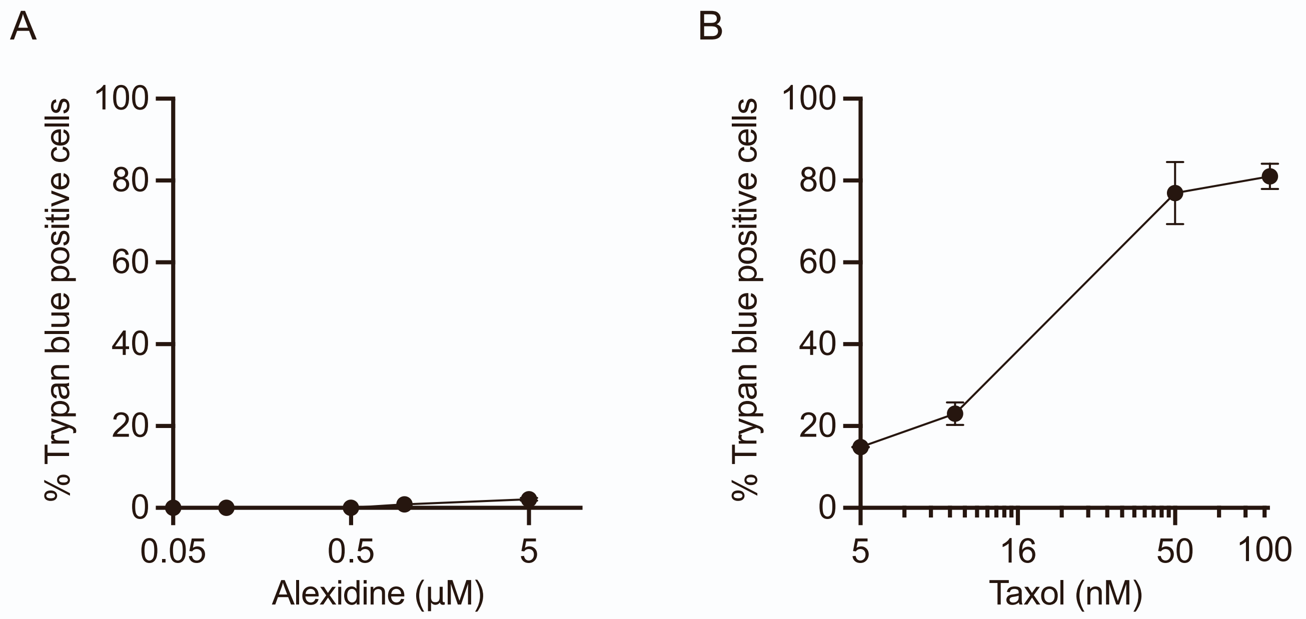

Figure S3. Non-specific cytotoxicity was not observed with alexidine treatment in non-tumorigenic breast cancer cells. The cytotoxic effects of alexidine (A) and Taxol (B) were examined in the non-tumorigenic breast cancer cell line MCF10A at increasing concentrations. The cells were treated with either alexidine (0, 0.05, 0.1, 0.5, 1, or 5 µM) or Taxol (0, 5, 10, 50 or 100 nM). After 48 hours of drug treatment, the percentage of cell deaths was determined by trypan blue. The data is presented as mean ± SD from three biological replicates (n = 3).

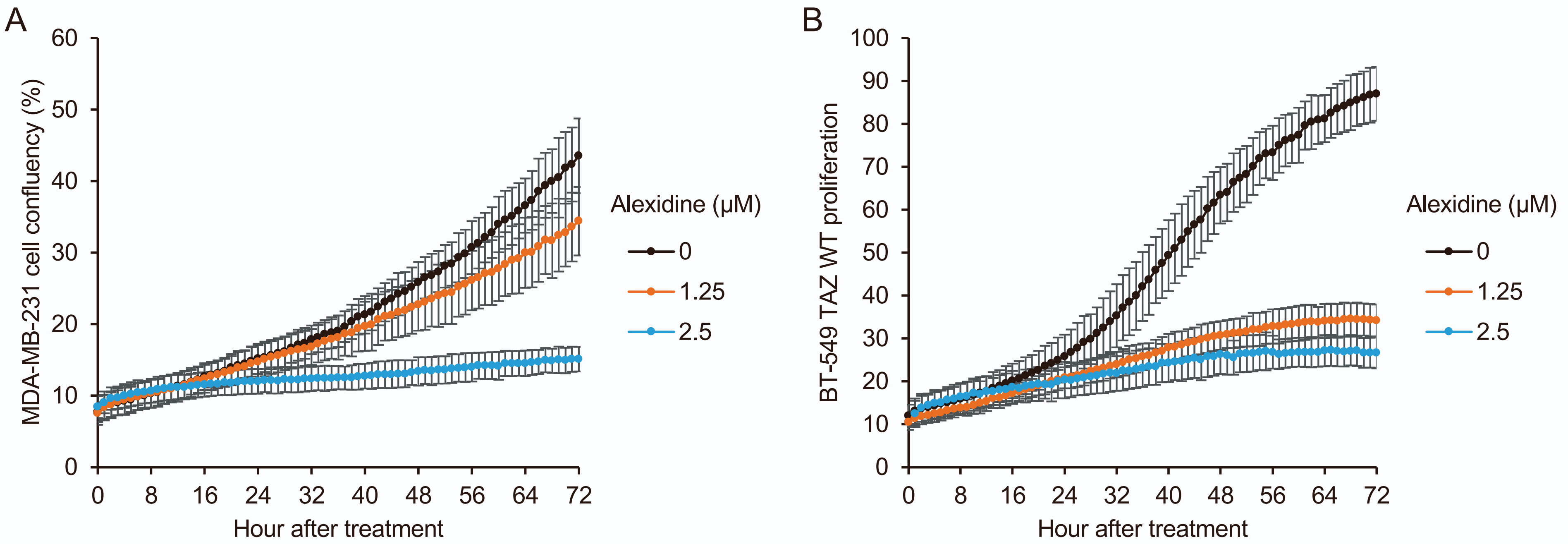

Figure S4. Alexidine inhibits the proliferation of breast cancer cells. The effect on cell proliferation was examined in MDA-MB-231 (A) and BT-549 (B) cells treated with different concentrations of alexidine. The cell confluency for each sample was monitored every hour using the CellCyte X machine for 3 days. The data is presented as mean ± SD from eight biological replicates (n = 8).

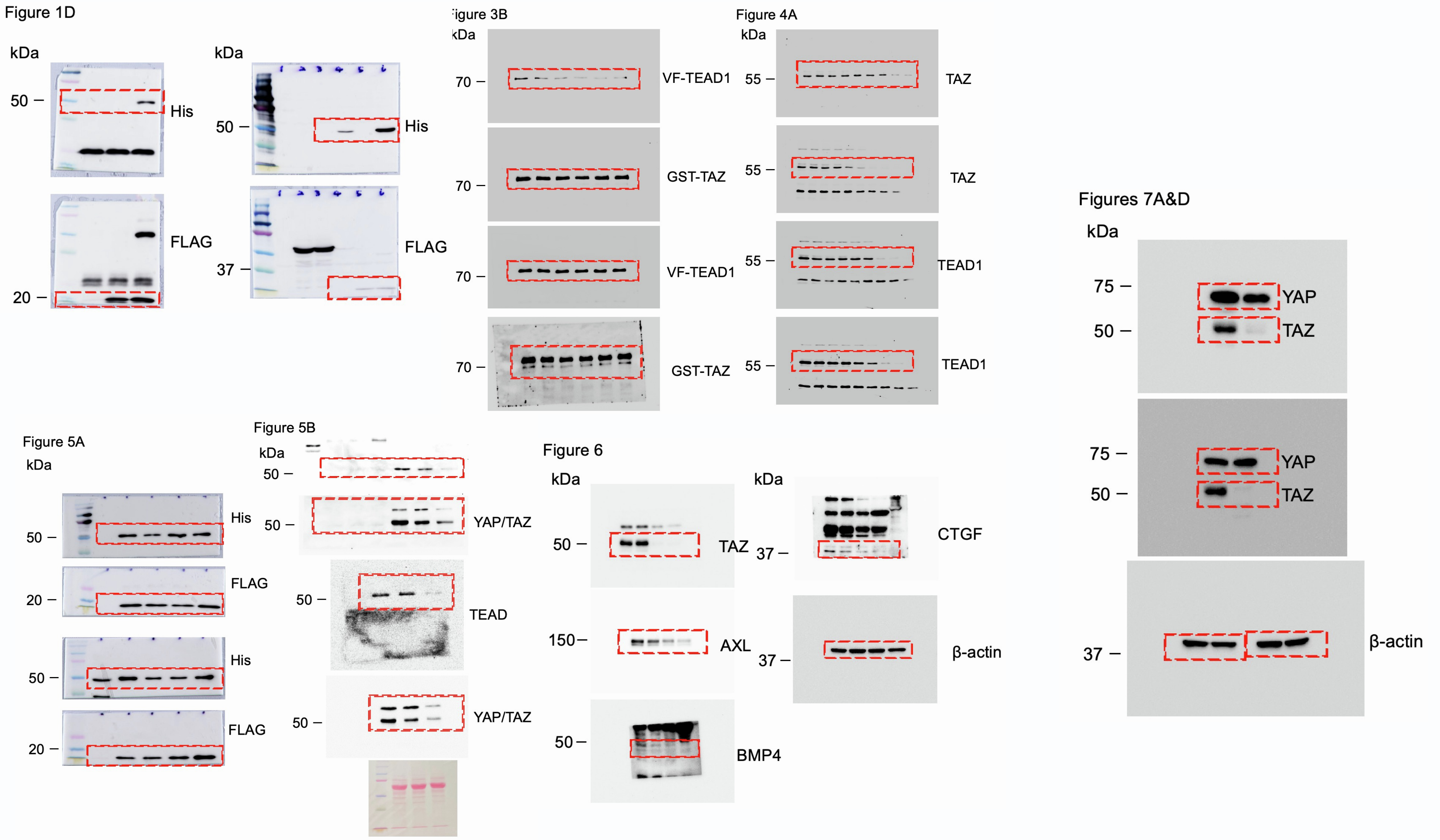

Figure S5. Uncropped, unedited WB images with one protein marker indicated.
