# Supplementary material for: Alexidine is a TAZ-specific small-molecule inhibitor that suppresses breast cancer invasion and metastasis
Source: iScience. 2025 Nov 21;28(12):114116. doi: 10.1016/j.isci.2025.114116 (PMC12719775; doi:10.1016/j.isci.2025.114116)
Supplement: Document S2. Table S1 [file mmc2.pdf]

Supplementary Table 1. Primary PDA-approved drug candidates from uHTS

	Drug name	CID	Target	Diseases	Brief description
1	Eltrombopag Olamine	135449331	Others	Cancer	a small molecule agonist of the c-mpl (TpoR) receptor
2	Bendamustine HCl	77082	DNA/RNA Synthesis	Cancer	inhibits SU-DHL-1 cell proliferation
3	Ponatinib (AP24534)	24826799	PDGFR,FGFR,VEGFR,Bcr-Abl	Cancer	a novel, potent multi-target inhibitor of Abl, PDGFRa, VEGFR2, FGFR1 and Src
4	Cisplatin	5460033	DNA/RNA Synthesis	Cancer	blocks DNA synthesis
5	Alfuzosin HCl	71764	Adrenergic Receptor	Cardiovascular Disease	an alpha1 receptor antagonist
6	Otilonium Bromide	72092	AChR	Cardiovascular Disease	an antimuscarinic
7	Bicalutamide	2375	Androgen Receptor	Endocrinology	An oral non-steroidal anti-androgen
8	Zinc Pyrithione	26041	Proton Pump	Infection	an antifungal and antibacterial agent
9	Thonzonium Bromide	11102		Infection	
10	Domiphen Bromide	10866	Others	Infection	a quaternary ammonium antiseptic
11	Colistin sulphate	71457944		Infection	
12	Oxytetracycline (Terramycin)	54675779	Others	Infection	the second of the broad-spectrum tetracycline group of antibiotics
13	Caspofungin Acetate	6850808	Others	Infection	a lipopeptide antifungal drug
14	Bazedoxifene HCl	9936012	Estrogen/progestogen Receptor	Metabolic Disease	a novel, non-steroidal, indole-based estrogen receptor modulator (SERM) binding to both ERa and ERß
15	Pergolide Mesylate	47812	Dopamine Receptor	Neurological Disease	an antiparkinsonian agent

16	Pimozide	16362		Neurological Disease	
17	Penfluridol	33630	Dopamine Receptor	Neurological Disease	a highly potent, first generation diphenylbutylpiperidine antipsychotic
18	Trifluoperazine 2HCl	66064	Autophagy	Neurological Disease	a dopamine D2 receptor inhibitor
19	Alexidine 2HCl	102678	Ptpmt1	Anti-bacteria/antifungal	Used in mouth wash
